# Acute inflammation triggered by two lightweight hernia meshes: a comparative in vitro and retrospective cohort study

**DOI:** 10.1007/s10029-025-03391-y

**Published:** 2025-06-17

**Authors:** Martin Reichert, Bernadet Massambo, Anca-Laura Amati, Veronika Grau, Katrin Richter, Andreas Hecker

**Affiliations:** 1 Department of General, Visceral, Thoracic and Transplant Surgery, Justus-Liebig University Giessen, University Hospital Giessen, Rudolf- Buchheim-Strasse 7, 35390 Giessen, Germany; 2 Laboratory of Experimental Surgery, German Center for Lung Research [DZL], Cardio-Pulmonary Institute [CPI], Justus-Liebig University Giessen, Feulgen-Strasse 10-12, 35385 Giessen, Germany

**Keywords:** Hernia repair, Lightweight mesh, Abdominal wall, Sublay, Inflammation, Retro-rectus

## Abstract

**Purpose:**

Retro-muscular mesh augmentation is standard for repairing abdominal incisional or larger primary hernia. A wide variety of meshes with diverse properties are available. The knowledge on the immune-modulating effects of meshes is, however, insufficient. This study investigates the impact of two widely used lightweight meshes, ULTRAPRO^®^ and ProGrip™, on macrophage activation (in vitro), systemic inflammation (in vivo), patient perioperative and long-term outcomes.

**Methods:**

Human THP-1 cell-derived macrophages were cultured in absence and presence of ULTRAPRO^®^ or ProGrip™ meshes. The release of pro-inflammatory cytokines, interleukin (IL)-1β and IL-6, was measured following inflammasome activation. In a retrospective study, systemic inflammation and postoperative outcomes after retro-muscular hernia repair using ULTRAPRO^®^ (321 patients) or ProGrip™ (161 patients) meshes were analyzed.

**Results:**

In the presence of ULTRAPRO^®^, IL-1β and IL-6 release by macrophages was increased, whereas ProGrip™ tended to reduce cytokine levels (*p* ≤ 0.05; *n* = 7). Baseline characteristics were comparable between both groups; systemic C-reactive protein levels were likewise higher in patients receiving ULTRAPRO^®^ compared to ProGrip™ (mean difference: 26.9 ± 7.5 mg/dl; *p* < 0.0001). No relevant differences were observed in perioperative morbidity or short-term outcomes, including complications and hospitalization after hernia repair, but hernia recurrence rates tended to be higher within three-year follow-up after ProGrip™ implantation compared to ULTRAPRO^®^ (*p* = 0.0630).

**Conclusion:**

Meshes exhibit distinct immune-modulating effects on macrophages, leading to differential activation that may influence foreign-body reaction and systemic inflammation. These immune responses potentially impact clinical outcomes and recurrence after hernia repair. This study underscores the need for comparative prospective, randomized-controlled trials to further evaluate the clinical relevance of mesh-specific immunological effects.

**Supplementary Information:**

The online version contains supplementary material available at 10.1007/s10029-025-03391-y.

## Introduction

Abdominal wall hernias require effective and long-lasting repair techniques that restore patient comfort and quality of life, sustaining these benefits in the long-term. The sublay technique, involving retro-muscular mesh augmentation, has become the gold standard for the treatment of larger, recurrent or incisional hernias [1]. This approach provides structural support to the weakened abdominal wall and reduces the risk of hernia recurrence [1–3]. Lightweight, large-pore, partially absorbable compound meshes are currently the widely used surgical standard delivering good clinical results [4, 5]. However, perioperative inflammation and hernia recurrence in the long-term follow-up are remaining concerns [2, 3, 5–10]. Various lightweight meshes are available, each with unique properties, and mesh selection often relies on surgeon preference or local standards. Although numerous clinical trials have recently investigated the use of innovative mesh products [11], comparative studies evaluating light-weight, large pore, and partially absorbable meshes are largely lacking. The impact of these meshes on early postoperative inflammation and long-term hernia recurrence rates after abdominal wall reconstruction needs to be studied in more detail.

When selecting the appropriate mesh, long-term hernia recurrence following sublay procedures should be considered, primarily due to defects in the integration of hernia meshes into the surrounding tissue. A rigid scar tissue around the mesh implant, however, is Janus-faced: while it reduces the risk of hernia recurrence, it is associated with significant draw-backs, such as the formation of a “stiff abdomen”, which adversely affects mobility, foreign body sensations, pain, and quality of life [5, 12, 13]. Adequate abdominal wall stability depends on a balanced scar formation, with collagen-rich fibrotic tissue integrating the mesh prosthesis into the surrounding tissue [2, 3, 5, 6].

Surgical hernia repair using meshes induces local trauma and acute inflammation with foreign body reaction, leading to the immigration of neutrophil granulocytes and monocytes that locally differentiate to macrophages [2, 3, 7, 14, 15]. Put in highly simplified terms, macrophages are classified into non-polarized M0-, pro-inflammatory M1- and anti-inflammatory M2-macrophages. In the early post-operative phase, M0-like macrophages differentiate towards M1-like macrophages that clear cellular debris and potential infections. M1-macrophages can also damage the surrounding tissue and the hernia mesh [16, 17]. The release of pro-inflammatory mediators like interleukin (IL)-1β and IL-6 by macrophages depend on local pro-inflammatory stimuli from traumatized tissue and the implanted mesh [2, 3, 6, 14, 15, 18, 19]. These pro-inflammatory mediators can enter the patient´s circulation and cause a systemic acute phase response, reflected by increased levels of acute-phase reactants like C-reactive protein (CRP) [14, 15, 20, 21].

Acute inflammation provokes a switch towards anti-inflammatory M2-like macrophages at the surgical site [2, 3, 14, 19, 22, 23]. M2-like macrophages orchestrate formation of connective tissue, which can result in appropriate wound healing and mesh integration, but also in excessive scar formation [5, 12, 18, 19, 24]. Of note, the extent of the initial acute inflammation significantly impacts on patient outcomes after sublay herniotomy, influencing scar formation and quality of tissue remodeling [2, 3, 5, 7, 18, 19, 24]. Therefore, a certain degree of inflammation might be essential for favorable outcomes, while too little or too much might be detrimental.

We hypothesize that the early local and systemic inflammatory response to sublay herniotomy depends, in part, on macrophage responses to the used mesh type. Early inflammation therefore may play a critical role in patient outcomes. Here, we investigated the effect of the two lightweight meshes ULTRAPRO^®^ (UP) and ProGrip™ (PG) on the release of the pro-inflammatory cytokines IL-1β and IL-6 by THP-1 cell-derived macrophages. Additionally, a single-center, retrospective clinical study compares early postoperative inflammatory responses and long-term clinical outcomes after sublay hernia repair using these lightweight meshes. We provide evidence that UP meshes both in vivo and in vitro, have a higher pro-inflammatory potential compared to PG. However, UP meshes might be associated with lower hernia recurrence rates.

## Methods

### In vitro experiments on THP-1 cell-derived macrophages

Monocytic THP-1 cells were obtained from the *German Collection of Microorganisms and Cell Cultures* (Braunschweig, Germany). These cells can be differentiated to macrophage-like cells and used for cytokine release experiments as described below. We did not use commercially available transfected reporter cells to directly visualize cell activation. In these cells, it may be difficult to discern signals caused by cell differentiation from signals induced by foreign surfaces. Cells were cultured in RPMI 1640 medium (Capricorn, Cat# RPMI-STA) supplemented with 10% FCS from Capricorn (Cat# FBS-16A). THP-1 cells were differentiated into macrophages in the absence or presence of UP (Partially Absorbable Lightweight Mesh, Ethicon, Johnson & Johnson Surgical Technologies) or PG (Self-gripping Mesh, Medtronic). UP meshes are composites of non-absorbable polypropylene (Prolene™) and resorbable copolymers of epsilon-caprolactone and glycolide (Monocryl™), while PG meshes are composed of a non-absorbable polyethylene terephthalate mesh with resorbable poly-lactic acid microgrips. The meshes were cut to a size of 0.5 cm^2^ under sterile conditions and fixed in 12-well plates (Greiner Bio-One, Frickenhausen, Germany) by using standard cannula (BD Microlance™ 3, 20G x1½” – Nr.1 [0.9 × 40 mm], Becton, Dickinson S.A.). As controls, cannula were placed in 12-well plates in the absence of the meshes. Both cannula and hernia meshes are expected to be sterile and free of endotoxins, because they are certified medical products.

A previously described protocol [25, 26] was used to differentiate THP-1 cells into M0-like or M1-like macrophages. In brief, 0.3 × 10^6^ monocytic THP-1 cells were seeded in 1 ml medium per well. The cells were treated with 50 nM PMA (Phorbol 12-myristate 13-acetate, Thermo Fisher Scientific, Cat# P1585) for 24 h, followed by incubation in complete medium without PMA for 24 h. Thereafter, cells were cultured in complete medium for additional 48 h to obtain the M0-like macrophages. Polarization to M1-like macrophages was performed in complete medium, supplemented with 10 ng/ml recombinant human interferon (IFN)-γ (R&D Systems, Minneapolis, MN, United States; Cat# 285-IF-100) and 10 ng/ml lipopolysaccharide (LPS, *E. coli* O111:B4, Merck, Cat# L2630) for 72 h.

On day 5 of differentiation, the medium was replaced by fresh complete medium, and the cells were left untreated or stimulated with 1 µg/ml LPS (*E. coli* O26:B6, Merck, Cat# L2654) and cultured for 5 h. Thereafter, the pore-forming toxin nigericin (50 µM; Merck, Cat# N7143) or adenosine 5′-triphosphate (ATP; 2 mM; Merck, Cat# A2383) was added for another 40 min. At the end of the experiments, cell culture supernatants were harvested, spun down (500 g, 8 min, 4 °C) and the cell-free supernatants were collected and stored at − 20 °C for later cytokine measurements. Cytokine concentrations were measured using the Human IL-1 beta/IL-1F2 DuoSet enzyme-linked immunosorbent assay (ELISA; R&D Systems, Cat# DY201-05) or the Human IL-6 DuoSet ELISA (R&D Systems, Cat# DY206-05).

### Clinical study

#### Patient data

This study was designed as a retrospective single-center cohort analysis to explore potential associations between different mesh types and systemic inflammatory responses. The retrospective approach was chosen to enable the integration of real-world clinical data with experimental findings and to serve as a hypothesis-generating basis for future prospective studies. The retrospective cohort study was approved by the local ethics committee of the University of Giessen medical faculty (No. 260/18) and performed in accordance with the latest version of the Declaration of Helsinki. All patients were treated according to the institutional standard-of-care. From 01/2009 to 12/2019 all consecutive patients who underwent abdominal wall hernia repair with mesh implantation using the retromuscular sublay technique were primarily included into the study. Patients who did not receive a UP or PG mesh were excluded from the data analysis (*n* = 15; Supplement 1).

Patient data were analyzed retrospectively from the prospectively maintained institutional database regarding general patient characteristics, characteristics of the hernia to be repaired and the surgical procedure. Postoperative complications were stratified according to the Clavien-Dindo classification of surgical complications [27] and summarized for further analysis using the Comprehensive Complication Index (CCI) [28]. Local seroma, hematoma and surgical site infections were additionally assessed. Medical records were reviewed until 2023 to identify patients developing hernia recurrence. Perioperative peak values of white blood cell count (WBC) and CRP levels were obtained on postoperative days (POD) 2 or 3 and POD 4.

#### Surgery and perioperative patient care

Abdominal wall hernia repair with mesh implantation by the retromuscular “sublay” technique are standard surgical procedures. Key principles of these procedures include resection of the hernia sac, extensive retromuscular dissection to create adequate space for mesh implantation. Meshes overlapped the former hernia defect or fascia incision, respectively, by at least 5 cm in all directions. From 2009 to 2016, the UP mesh was used. From 2017 onwards, the clinical standard was changed to the PG mesh. This change was not based on patient-specific criteria but reflected a shift in surgical preference due to the handling characteristics and self-fixating properties of the PG mesh, which offered potential advantages in mesh placement and fixation. Thus, patient allocation to either UP or PG was based solely on the time of surgery within the defined study period. During surgery, the peritoneum or the posterior leaf of the rectus sheath, respectively, was closed using slowly absorbable running suture (1 [4 Ph. Eur.] PDS™, Polydioxanone, Ethicon^®^). This forms the dorsal mesh layer, onto which the meshes are applied in a size-adapted manner. UP meshes were fixed with Prolene™ 2 − 0 (3 Ph. Eur. Polypropylen, Ethicon^®^) single sutures at the edges to the posterior mesh layer. Due to their self-gripping properties, PG meshes were placed on the dorsal mesh bed and were not additionally secured. Fascial closure was achieved using non-absorbable running suture (1 [4 Ph. Eur.] Prolene™, Ethicon^®^). Postoperatively, patients were treated by principles of a “fast track” protocol including early extubation, early enteral nutrition and early mobilization [29–33].

### Statistical analyses

Statistical analyses were performed using GraphPad Prism (Version 9, GraphPad Software, San Diego, CA, USA) or SPSS (IBM SPSS Statistics for Windows, Version 24.0). For all in vitro experiments, seven biological replicates were performed using different passages of the cells, each.

For Figs. 2 and 3, cytokine concentrations in cell culture supernatants of controls were set to 100% and all other data were calculated accordingly. Results of cytokine release from cell culture experiments (*n* = 7, each) were analyzed using the Friedman test followed by the Wilcoxon signed-rank test, if applicable. Data are presented in the boxplots as individual data points, bars represent median, whiskers extend from 25th to 75th percentile. No outliers were excluded from the analyses. The data were visualized using Inkscape version 0.48.5 r10040 (Free and Open Source Software licensed under the GPL).

The patient cohort was divided into the two groups UP and PG meshes. For descriptive statistics, categorical data of both groups were analyzed using Fisher’s exact test. Given the retrospective nature of the study and unequal group sizes, we did not perform formal testing for normality. Instead, the more rigorous non-parametric tests were applied to account for potential deviations from normal distribution. Two-group comparisons of continuous variables were performed by Mann-Whitney-U test. Intra-group longitudinal comparisons based on UP or PG mesh sizes were conducted using Kruskal-Wallis test for global effects, and, if applicable, followed by Dunn´s test, adjusted for multiple comparisons. Bars in boxplots depict median, whiskers indicate minimum to maximum ranges, and the boxes extend from the 25th to 75th percentiles. Data are given in tables as medians with interquartile ranges for continuous variables as well as n (%) for categorical variables.

Cumulative incidences of hernia recurrence were calculated over a three-year follow-up period, starting from the date of index hernia repair, using Kaplan-Meier estimation up to week 160 after index surgery. Kaplan-Meier curve comparisons were performed by Gehan-Breslow-Wilcoxon test. Patients in whom no hernia recurrence was described within the three-year follow-up or patients who were lost to follow-up were censored from the analysis of cumulative incidences upon their last contact. Vertical ticks in the figure indicate censored data.

To determine statistical dependences between mesh sizes and postoperative CRP, simple linear regression was applied. To identify independent factors influencing postoperative inflammatory response, objectified by systemic CRP peak elevation on POD 2 or 3, a stepwise approach was used. Clinically relevant parameters were first assessed using univariable linear regression, and variables with p-values ≤ 0.01 were subsequently included in a multivariable linear regression model. For linear regression analyses, assumptions of independence, linearity, and homoscedasticity were considered. As each patient contributed only one data point, independence of observations was ensured. Given the retrospective and exploratory nature of the study, no formal diagnostic tests for linearity or homoscedasticity were performed.

P-values ≤ 0.05 indicate statistical significance. A trend or tendency is defined by p-values below 0.1 but above 0.5.

## Results

### Cytokine release by THP-1-derived macrophages

#### Cytokine release in the absence of meshes

**Fig. 1:**
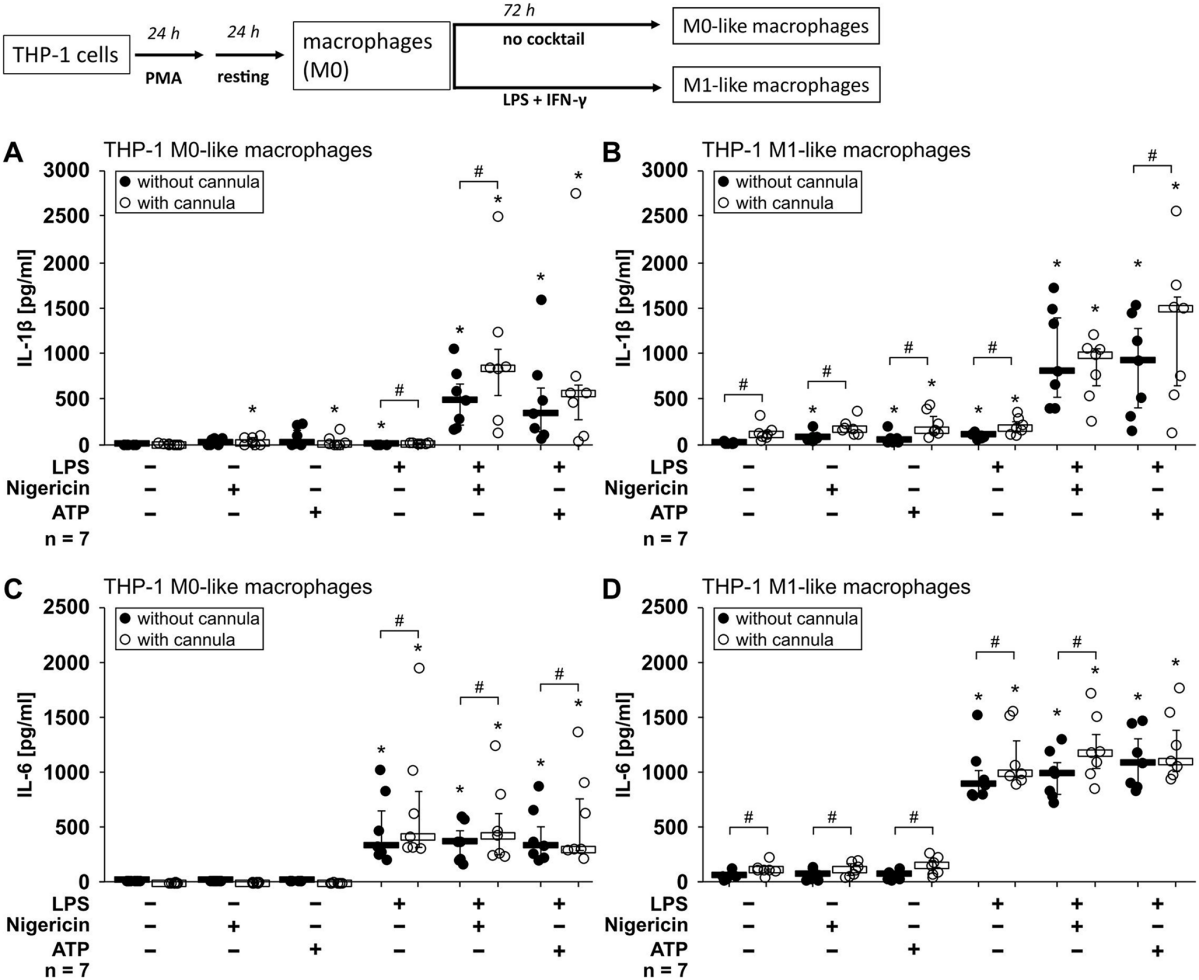
Interleukin (IL)-1β and IL-6 release by macrophage-like THP-1 cells. THP-1 cells were differentiated to macrophage-like cells (**A**,**C**; M0-like) using phorbol 12-myristate 13-acetate (PMA) in the absence (without cannula) and presence (with cannula) of the cannula. (**B**) Macrophage-like cells were further differentiated into a M1-like phenotype using lipopolysaccharide (LPS) and interferon-γ (IFN-γ; **B**, **D**). On day 5 of differentiation, macrophage-like cells were left untreated or primed with LPS (1 µg/ml) for 5 h. Thereafter, the pore-forming toxin nigericin (50 µM) or ATP (2 mM) was added for another 40 min to trigger inflammasome assembly. The concentrations of IL-1β (**A**, **B**) and IL-6 (**C**, **D**) were measured in cell culture supernatants by ELISA. Seven biological replicates were performed each (*n* = 7), using different passages of the cell line. Data are presented as individual data points, bars represent median, whiskers percentiles 25 and 75. Friedman test followed by the Wilcoxon signed-rank test. ∗*p* ≤ 0.05 significantly different from corresponding untreated control samples. # *p* ≤ 0.05 significantly different

In the early phase of a foreign-body reaction, pro-inflammatory cytokines secreted by innate immune cells are expected to be center stage. Some of the cytokines are secreted in response to a single stimulus indicating danger. Other cytokines are produced as a pro-form and need a second danger signal that leads to the assembly of an inflammasome, a multi-protein complex that is essential for cytokine maturation and release. We first analyzed the LPS-induced inflammasome-independent release of IL-6 [34, 35] and the inflammasome-dependent release of IL-1β [36] in the absence of meshes. Monocytic THP-1 cells, differentiated towards M0-like and M1-like macrophages, were either left untreated or primed with LPS (1 µg/ml, 5 h). As second danger signals ATP (2 mM) or the pore-forming toxin nigericin (50 µM) were added for 40 min to induce IL-1β release [36–38]. In the absence of any stimulus, M0-like and M1-like macrophages released small amounts of IL-1β throughout, while nigericin induced a slight but significant increase in M0-like macrophages (Fig. 1A, B). ATP slightly increased the IL-1β release by M0- and M1-like macrophages (Fig. 1A, B). LPS priming alone caused a slight increase in IL-1β levels in M1-like THP-1 cell supernatants β (Fig. 1B). As expected, a considerable increase in IL-1β release was seen in LPS-primed M0- and M1-like macrophages, when treated with nigericin or ATP (Fig. 1A, B). Priming with LPS resulted in a strong increase of IL-6 levels (Fig. 1C, D). As expected for an inflammasome-independent cytokine, neither addition of nigericin nor of ATP further increased the secretion of IL-6 (Fig. 1C, D). To fix hernia meshes on the bottom of the wells of cell culture plates, cannula were used. The presence of these cannula slightly impacted cytokine releases under some conditions (Fig. 1).

#### Effects of hernia meshes on IL-1β release

**Fig. 2:**
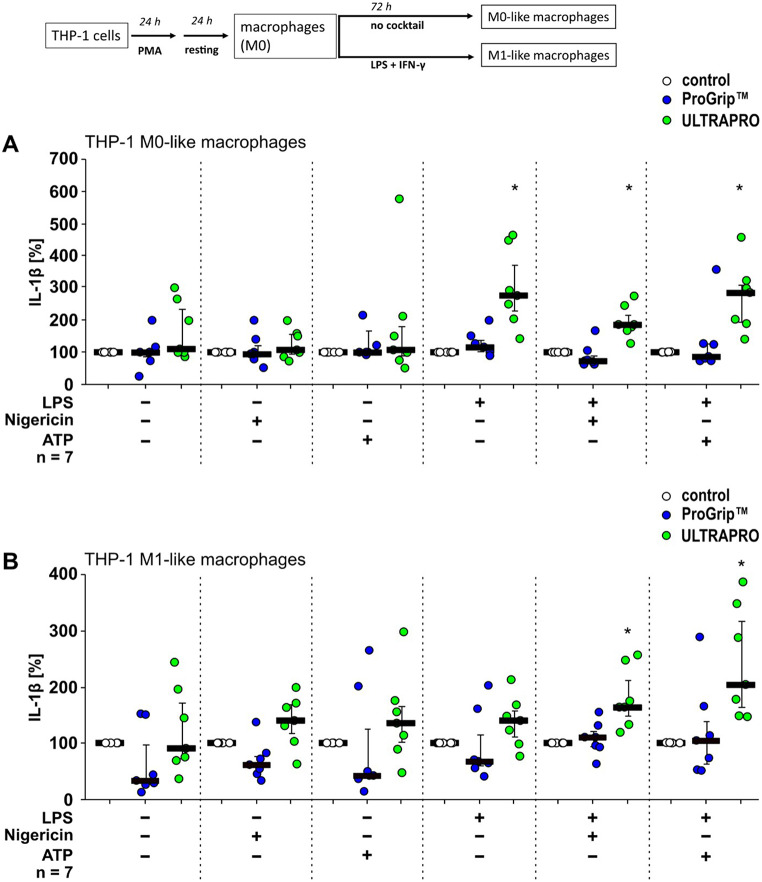
ULTRAPRO^®^ meshes enhance interleukin (IL)-1β release by macrophage-like THP-1 cells. THP-1 cells were seeded in the absence (control, white circles) or the presence of hernia meshes (ProGrip^™^, blue circles; ULTRAPRO^®^, green circles; 1 cm^2^ mesh per well) and differentiated to macrophage-like cells (**A**; M0-like) using phorbol 12-myristate 13-acetate (PMA). (**B**) Macrophage-like cells were further differentiated into a M1-like phenotype using lipopolysaccharide (LPS) and interferon-γ (IFN-γ; **A**, **B**) On day 5 of differentiation, macrophage-like cells were left untreated or primed with LPS (1 µg/ml) for 5 h. Thereafter, the pore-forming toxin nigericin (50 µM) or ATP (2 mM) was added for another 40 min to trigger IL-1β release. Seven biological replicates were performed each (*n* = 7), using different passages of the cell line. The IL-1β concentration in supernatants of control cells was set to 100% and all other data were normalized accordingly. Data are presented as individual data points, bars represent median, whiskers percentiles 25 and 75. Friedman test followed by the Wilcoxon signed-rank test. ∗*p* ≤ 0.05 significantly different from corresponding untreated control samples

**Fig. 3:**
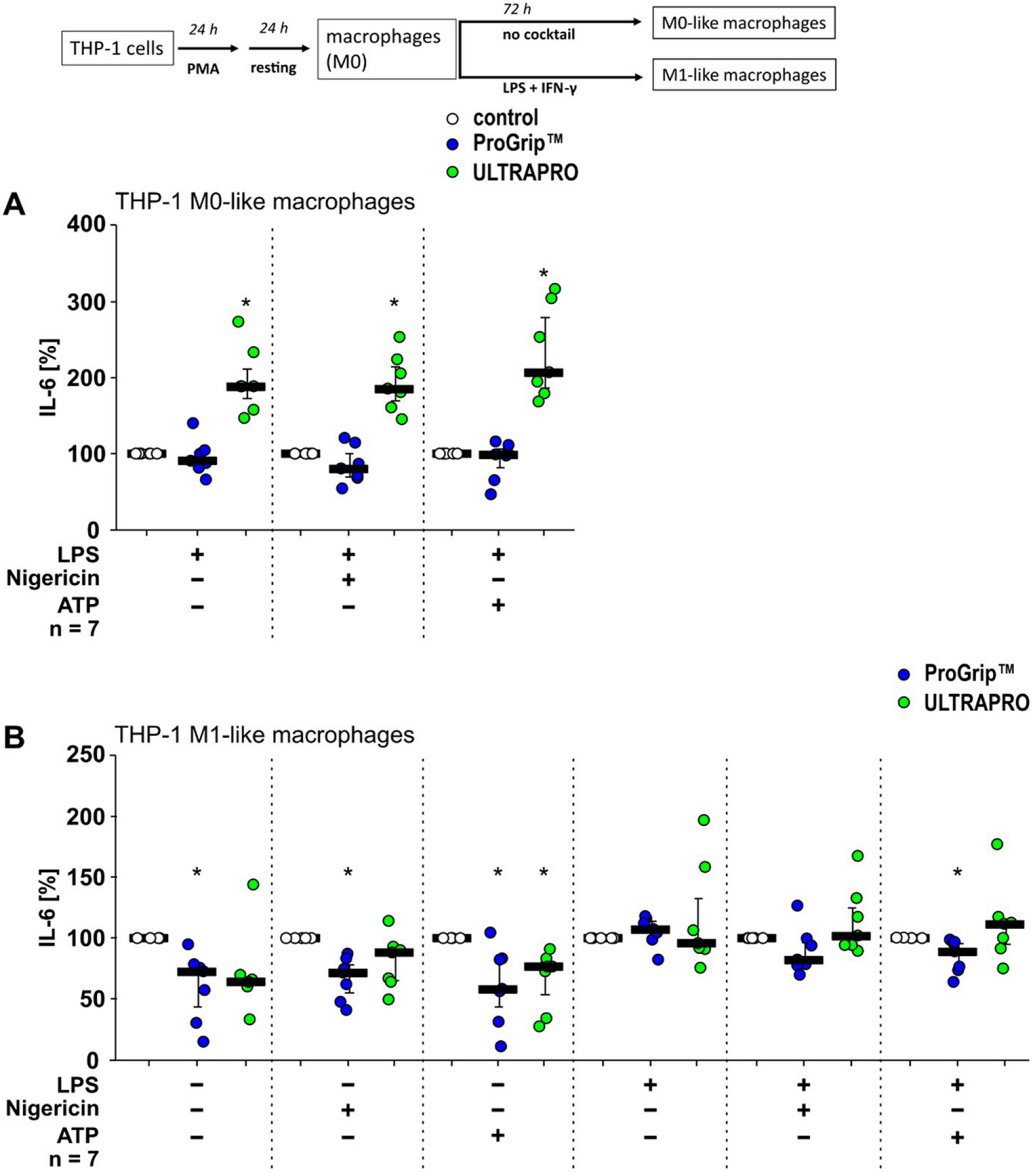
ProGrip^™^ and ULTRAPRO^®^ meshes modulate interleukin (IL)-6 release by macrophage-like THP-1 cells. THP-1 cells were seeded in the absence (control, white circles) or the presence of hernia meshes (ProGrip^™^, blue circles; ULTRAPRO^®^, green circles; 1 cm^2^ mesh per well) and differentiated to macrophage-like cells (**A**; M0-like) using phorbol 12-myristate 13-acetate (PMA). (**B**) Macrophage-like cells were further differentiated into a M1-like phenotype using lipopolysaccharide (LPS) and interferon-γ (IFN-γ; **A**, **B**) On day 5 of differentiation, macrophage-like cells were left untreated or primed with LPS (1 µg/ml) for 5 h. Thereafter, the pore-forming toxin nigericin (50 µM) or ATP (2 mM) was added for another 40 min and the IL-6 concentration was determined in cell-free supernatants by ELISA. Of note, in supernatants of M0-like cells the IL-6 concentrations in the absence of LPS were below the limit of detection. Both meshes enhance IL-6 release by LPS-primed M0-like cells. ProGrip^™^ and ULTRAPRO^®^ seems to reduce IL-6 release by M1-like cells in the absence of LPS. Seven biological replicates were performed each (*n* = 7), using different passages of the cell line. The IL-6 concentration in supernatants of control cells was set to 100% and all other data were normalized accordingly. Data are presented as individual data points, bars represent median, whiskers percentiles 25 and 75. Friedman test followed by the Wilcoxon signed-rank test. ∗*p* ≤ 0.05 significantly different from corresponding untreated control samples

Next, IL-1β release by THP-1 cells differentiated in the presence of PG or UP meshes was compared to that of cells without contact to meshes (Fig. 2). For better visualization, the cytokine levels obtained in the same experimental setting (with cannula) but without meshes (Fig. 1) were set to 100%. The cytokine levels obtained in the presence of UP or PG were calculated accordingly (Figs. 2 and 3). When M0-like macrophages were not primed with LPS, both mesh types did not change the amount of IL-1β released into the supernatant (Fig. 2). Upon priming with LPS (1 µg/ml), however, the presence of UP mesh markedly increased IL-1β release, while PG meshes did not (Fig. 2A). The same was true when LPS-primed M0-like macrophages were further stimulated with nigericin (50 µM) or ATP (2 mM; Fig. 2A). The concentration of IL-1β in the supernatant of M1-like macrophages in the presence of PG showed a tendency to decrease (*p* = 0.063; Fig. 2B). In contrast, UP significantly increased IL-1β release in LPS-primed cells stimulated with nigericin or ATP (*p* ≤ 0.05; Fig. 2B). A tendency in the same direction was seen, when cells were treated with nigericin (*p* = 0.063; Fig. 2B) or LPS alone (*p* = 0.091; Fig. 2B). Taken together, PG tended to reduce the release of IL-1β under certain conditions, while in the presence of UP significantly more IL-1β was released in an inflammasome-dependent manner.

#### Effects of hernia meshes on IL-6 release

The presence of PG did not significantly alter the release of IL-6 by M0-like macrophages (Fig. 3A). UP increased IL-6 release in LPS-primed cells (*p* ≤ 0.05; Fig. 3A), a response also observed after stimulation with nigericin or ATP (Fig. 3A). In unprimed M1-like macrophages, PG reduced the release of IL-6 (*p* ≤ 0.05; Fig. 3B), whereas it had no relevant effect on cells primed with LPS.

### Clinical data

#### General characteristics of the patient cohort

**Fig. 4:**
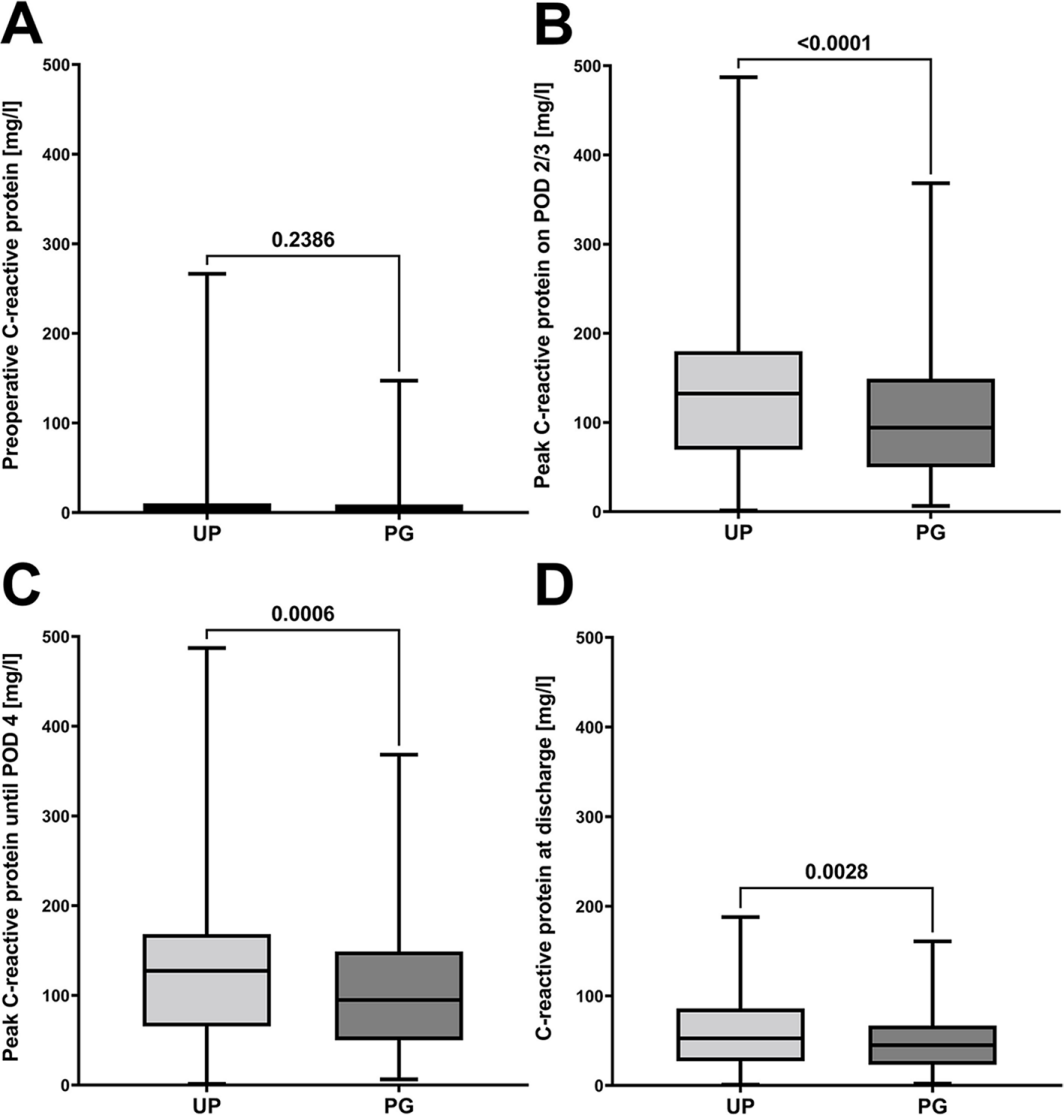
Perioperative systemic C-reactive protein values. Differences in systemic C-reactive protein values after hernia repair using the sublay technique between ULTRAPRO^®^ (UP) and ProGrip™ (PG) meshes. **A**: preoperative systemic C-reactive protein values. **B**: systemic peak C-reactive protein values on postoperative days (POD) 2 or 3. **C**: systemic peak C-reactive protein values until postoperative day 4. D: systemic C-reactive protein values at discharge. Data are presented as boxplots, where the boxes represent the interquartile range (25th to 75th percentiles), the horizontal line indicates the median, and whiskers show the minimum and maximum values. Comparisons between two groups were performed using the Mann–Whitney U test. Results are reported with the corresponding p-values, and *p* ≤ 0.05 was considered statistically significant

Between 01/2009 and 12/2019, 482 patients met the inclusion criteria and underwent open hernia repair and abdominal wall reconstruction using the retromuscular sublay mesh repair technique with UP or PG meshes. Until 12/2016 UP was used in 321 patients, and 161 patients obtained PG meshes since 01/2017. Patient characteristics were widely balanced between the UP and PG patient cohort including chronic diseases such as diabetes that might impair wound healing (Table 1). No differences were observed in preoperative CRP values nor WBC in peripheral blood (Fig. 4A, Supplement 2 ). Most surgical procedures were performed electively, and the most common indication was median incisional hernia (Table 1).

**Table 1:** Characteristics of patients, hernia and surgical procedures. Categorical data of both groups were compared using fisher’s exact test. Two-group comparisons of continuous variables were performed by Mann-Whitney-U test. data are given as median and interquartile range or n (%). $ including coronary artery disease. * excluding patients with stable transplant kidney function. # including HIV, chemotherapy six weeks prior to surgery, or history of (solid) organ transplantation. § data not available retrospectively in 10 patients from the ULTRAPRO^®^-group. BMI = body mass index. ASA = American society of anesthesiologist´s classification of physical health (ASA) score

Variable	ULTRAPRO^®^ (*n* = 321)	ProGrip™ (*n* = 161)	*p*-value
**Patient characteristics**			
Female gender [*n patients*]	146 (45.5%)	60 (37.3%)	0.0971
Age [*years*]	63 (53.5–72)	61 (52–71)	0.1936
BMI [*kg/m²]*	28.5 (24.9–32.0)	28.9 (25.6–32.3)	0.8040
ASA [*score*] 1 [*n patients*] 2 [*n patients*] 3 [*n patients*] 4 [*n patients*]	3 (2–3) 7 (2.2%) 150 (46.7%) 152 (47.4%) 12 (3.7%)	2 (2–3) 2 (1.2%) 86 (53.4%) 70 (43.5%) 3 (1.9%)	0.2324
Chronic diseases [*n patients*]	236 (73.5%)	106 (65.8%)	0.0890
Charlson Comorbidity Index [points]	2 (1–3)	2 (0–4)	0.9651
Arterial hypertension [*n patients*]	217 (67.6%)	94 (58.4%)	0.0550
Chronic cardiac disease [*n patients*] ^$^	106 (33.0%)	41 (25.5%)	0.0942
Chronic pulmonal disease [*n patients*]	53 (16.5%)	21 (13.0%)	0.3506
Diabetes mellitus [*n patients*]	69 (21.5%)	31 (19.3%)	0.6343
Chronic kidney disease [*n patients*] *	55 (17.1%)	19 (11.8%)	0.1415
Chronic liver disease [*n patients*]	22 (6.9%)	9 (5.6%)	0.6959
Systemic immunosuppression [*n patients*] ^#^	32 (10.0%)	21 (13.0%)	0.3545
Peripheral artery disease [*n patients*]	16 (5.0%)	9 (5.6%)	0.8286
Previous malignoma [*n patients*]	107 (33.3%)	63 (39.1%)	0.2258
Active smoking [*n patients*]	99 (30.8%)	43 (26.7%)	0.3970
Active alcohol abuse [*n patients*]	49 (15.3%)	19 (11.8%)	0.3339
Previous abdominal surgery [*n patients*]	289 (90.0%)	147 (91.3%)	0.7435
**Hernia characteristics**			
Elective surgery [*n patients*] Emergency surgery	296 (92.2%) 25 (7.8%)	154 (95.7%) 7 (4.3%)	0.1776
Primary hernia [*n patients*] Secundary (incisional) hernia	36 (11.2%) 285 (88.8%)	18 (11.2%) 143 (88.8%)	1
Recurrent hernia [*n patients*]	63 (19.6%)	24 (14.9%)	0.1392
Previous mesh implantation [*n patients*]	32 (10.0%)	12 (7.5%)	0.2447
Isolated medial hernia [*n patients*] Isolated lateral hernia Combined medial and lateral hernia	259 (80.7%) 26 (8.1%) 36 (11.2%)	141 (87.6%) 5 (3.1%) 15 (9.3%)	0.0767
**Procedure characteristics**			
Duration of surgery [*min*]	106.0 (79.0-151.5)	117.0 (82.0-149.5)	0.2114
Mesh size [*cm²*] ^§^	450 (225–600)	450 (300–450)	0.9552
Relevant adhesiolysis [*n patients*]	184 (57.3%)	102 (63.4%)	0.2381
Relevant additional procedures [*n patients*]	74 (23.1%)	44 (27.3%)	0.3137
Additional mesh implantation [*n patients*] Inguinal hernia repair Parastomal hernia repair Hiatal hernia repair Open intraperitoneal onlay mesh	11 (3.4%) 3 1 3 4	10 (6.2%) 6 1 3 1	0.1632
Major liver resection [*n patients*] Minor liver resection	5 (1.6%) 0	2 (1.2%) 1 (0.6%)	1
Cholecystectomy [*n patients*]	8 (2.5%)	9 (5.6%)	0.1137
Lymphnode exstirpation / dissection [*n patients*]	5 (1.6%)	12 (7.5%)	0.0025
Omentectomy [*n patients*]	10 (3.1%)	2 (1.2%)	0.3528
Relevant gastrointestinal surgery [*n patients*] Upper gastrointestinal surgery Small bowel surgery Large bowel surgery Stoma revision	23 (7.2%) 3 6 11 3	10 (6.2%) 0 2 7 1	0.8487

#### Surgical procedures

No significant differences were observed in the frequency of major surgical procedures performed together with hernia repair, specifically regarding potentially contaminated gastrointestinal surgery. In addition, no differences were seen in the duration of surgery as well as in the size of UP or PG meshes implanted during sublay procedure (Table 1).

#### Postoperative systemic inflammation and patient outcome

**Fig. 5:**
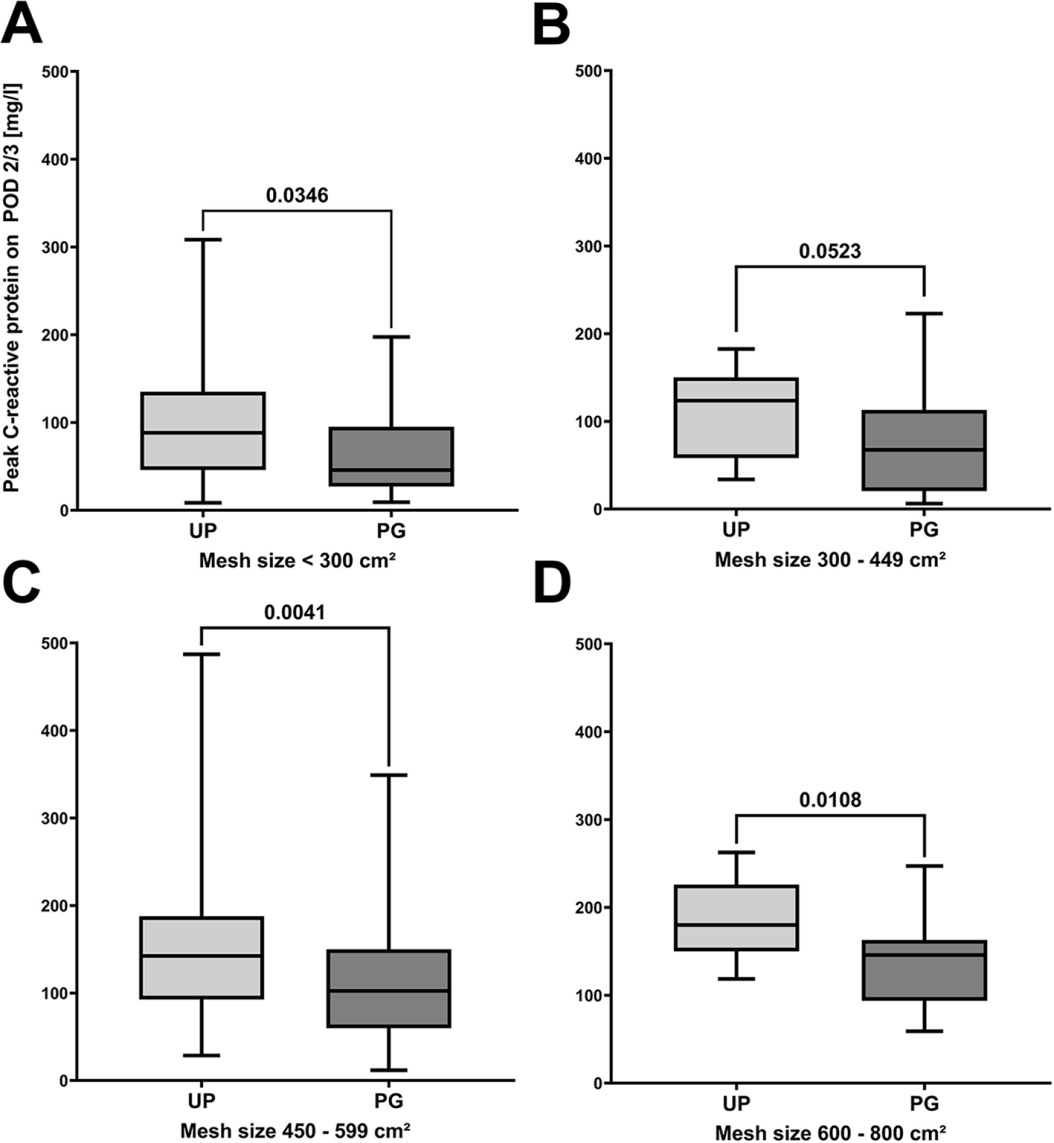
Mesh size related postoperative systemic inflammation. Differences in postoperative peak systemic C-reactive protein values after hernia repair using the sublay technique between ULTRAPRO^®^ (UP) and ProGrip^™^ (PG) meshes related to the cumulative size of mesh implants. **A**-**D**: systemic peak C-reactive protein on postoperative days (POD) 2 or 3. Mesh size was stratified from < 300 cm² to 800 cm². Data are presented as boxplots, where the boxes represent the interquartile range (25th to 75th percentiles), the horizontal line indicates the median, and whiskers show the minimum and maximum values. Comparisons between two groups were performed using the Mann–Whitney U test. Results are reported with the corresponding p-values, and *p* ≤ 0.05 was considered statistically significant

**Fig. 6:**
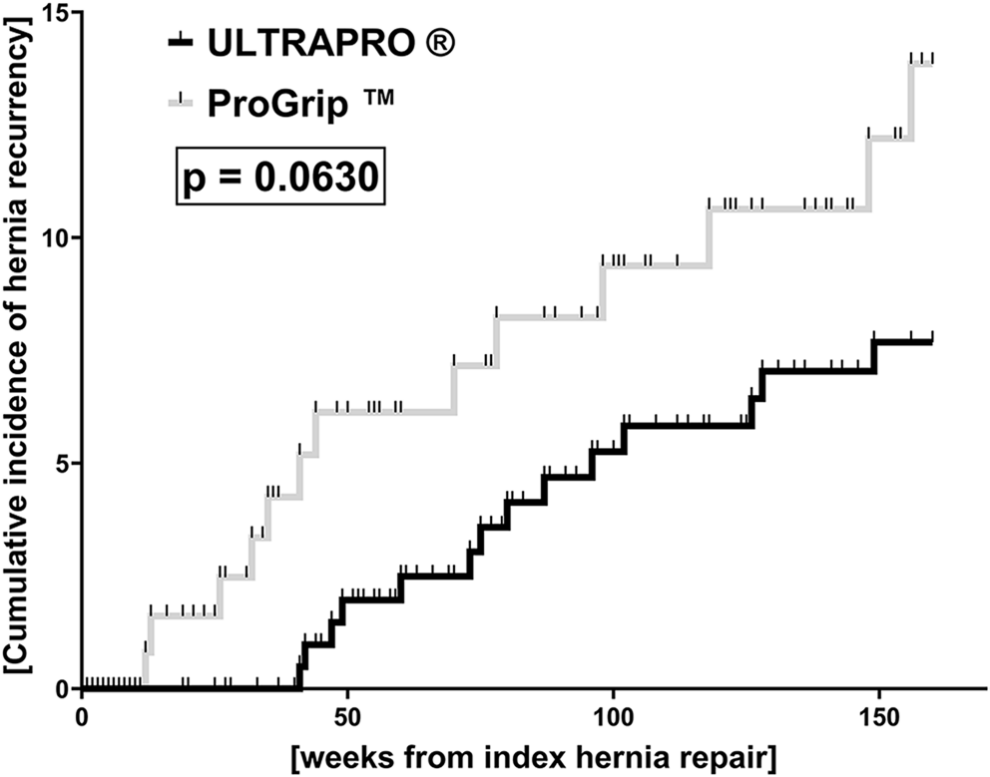
Cumulative incidence of hernia recurrence within three years after index hernia repair by using retromuscular mesh implantation. Cumulative incidence of hernia recurrence tended to be higher in patients who underwent hernia repair with retromuscular ProGrip™ mesh augmentation during the observational period of 160 weeks after index surgery (*p* = 0.0630 in Gehan-Breslow-Wilcoxon test)

No differences occurred in rate of seroma, in the rate of overall complications stratified by severity [27], in the comprehensive complication index [28] between both groups during the postoperative in-hospital stay (Table 2). Although surgical trauma and mesh sizes were similar, CRP values were markedly increased in the UP compared to the PG cohort on POD 2/3 (mean difference: 26.9 ± 7.5 mg/dl), until POD 4 (mean difference: 22.3 ± 7.3 mg/dl) and on the day of discharge (mean difference: 12.9 ± 3.8 mg/dl; Fig. 4). These global differences remained consistent after stratifying the two cohorts based on mesh sizes (Fig. 5). However, no differences were seen in perioperative WBCs (Supplement 2). Median follow-up was not significantly different between both groups (Table 2). However, in the long-term follow-up, an increase in the cumulative incidence of hernia recurrence was observed, with a non-significant trend toward higher recurrence rates in patients receiving PG implants compared to UP (*p* = 0.0630; Fig. 6).

**Table 2:** Postoperative morbidity. Postoperative complications were classified according to the Clavien-Dindo classification of surgical complications [27]. Morbidity was summarized using the comprehensive complication index, which is based on the Clavien-Dindo classification [28]. Categorical data of both groups were compared using fisher’s exact test. Two-group comparisons of continuous variables were performed by Mann-Whitney-U test. Data are given as median and interquartile range or n (%). * excluding re-operations. PO = postoperative. POD = postoperative day

Variable	ULTRAPRO^®^ (*n* = 321)	ProGrip™ (*n* = 161)	*p*-value
PO length of in-hospital stay [d]	5 (4–7)	5 (4–7)	0.2220
PO stay at intensive care unit [*n patients*]	64 (19.9%)	24 (14.9%)	0.2114
Re-intubation [*n patients*]*	11 (3.4%)	3 (1.9%)	0.4032
PO secondary antibiotic therapy until POD 30 [*n patients*]	106 (33.0%)	36 (22.4%)	0.0196
Overall median follow-up [weeks from index surgery]	114 (1-288)	98 (16–189)	0.1590
Median 3-year follow-up [weeks from index surgery]	114 (1-160)	97 (16–160)	0.8207
**Postoperative complications**			
Any postoperative complication [n patients]	114	62	0.5479
Superficial surgical site infections [n patients]	38	19	1
Overall seroma rate [n patients]	62	39	0.2357
Seroma without treatment [n patients]	21	17	0.1510
Seroma needing treatment [n patients]	41	22	0.7762
Mesh infection requiring mesh explantation [n patients]	1	0	1
Comprehensive complication index [score]	0 (0-20.9)	0 (0-20.9)	0.5138
Grade I [n patients]	40	16	
Grade II [n patients]	64	38	
Grade IIIa [n patients]	32	16	
Grade IIIb [n patients]	25	18	
Grade IVa [n patients]	4	2	
Grade IVb [n patients]	4	0	
30-day mortality [n patients]	4 (1.3%)	2 (1.2%)	

#### Regression and multivariable analyses

Simple linear regression revealed a positive correlation between mesh size and postoperative CRP levels for both mesh types (Supplement 3). In the multivariable linear regression model, mesh type remained an independent predictor of systemic CRP peak levels on POD 2 or 3, alongside other surgery-related factors (Table 3). These findings support an association between mesh type and the extent of early postoperative systemic inflammation.

**Table 3:** Perioperative determinants of systemic inflammation. Perioperative variables influencing systemic peak C-reactive protein values on postoperative days 2 or 3 were evaluated by univariable and multivariable analysis. Notably, the type of implanted mesh significantly impacts the extent of systemic inflammation. While ULTRAPRO^®^ markedly triggers systemic inflammation, ProGrip^TM^ has a reducing impact on peak C-reactive protein values on postoperative days 2 or 3. BMI = body mass index. CCI = Charlson comorbidity index. WBC = white blood cell count. CRP = C-reactive protein. POD = postoperative day. UP = ULTRAPRO^®^. PG = ProGrip^TM^. SSI = surgical site infection. * including operations on the Gastrointestinal tract

Variable	Univariable analysis			Multivariable analysis				
	*r* ^2^	95% CI	*p* value	Coefficients			t	*p* value
				B	Std. error	95% CI		
**Peak C-reactive protein on postoperative day 2 or 3**								
Age [years]	0.02981	0.014–0.047	**0.0003**	0.6556	0.2369	0.1897–1.121	2.767	**0.0059**
BMI [kg/m²]	0.01409	0.002–0.019	0.0139	**-**	**-**	**-**	**-**	**-**
CCI [points]	0.01057	0.000-0.006	0.0333	**-**	**-**	**-**	**-**	**-**
Emergency surgery	0.03780	0.000-0.001	**< 0.0001**	31.58	14.82	2.435–60.72	2.131	**0.0338**
Secondary hernia	0.00678	-0.000-0.001	0.0888	**-**	**-**	**-**	**-**	**-**
Preoperative WBC [giga/l]	0.04143	0.005–0.012	**< 0.0001**	0.3703	1.312	-2.209-2.949	0.2823	0.7779
Preoperative CRP [mg/l]	0.09648	0.078–0.145	**< 0.0001**	0.3204	0.1354	0.05408–0.5866	2.366	**0.0185**
Peak WBC on POD 2/3	0.14280	0.014–0.022	**< 0.0001**	4.499	1.132	2.273–6.726	3.973	**< 0.0001**
**Type of mesh [UP]**	0.2958	0.001–0.002	**0.0003**	21.93	6.480	9.186–34.67	3.384	**0.0008**
**Type of mesh [PG]**	-0.0295	-0.002- -0.001	**0.0004**	-24.04	6.585	-36.99- -11.10	3.651	**0.0003**
Size of mesh [cm²]	0.1049	0.724–1.290	**< 0.0001**	0.04922	0.01483	0.02006–0.0784	3.319	**0.0010**
Potentially contaminated surgery *	0.05635	0.001–0.001	**< 0.0001**	7.489	11.40	-14.93-29.90	0.6569	0.5116
Adhesiolysis	0.03200	0.001–0.002	**0.0002**	10.21	6.839	-3.237-23.66	1.493	0.1363
Duration of surgery [min]	0.1717	0.276–0.422	**< 0.0001**	0.3319	0.0605	0.2129–0.4510	5.483	**< 0.0001**
Postoperative SSI	0.01093	0.000-0.001	0.0314	**-**	**-**	**-**	**-**	**-**

## Discussion

This study demonstrates that UP meshes enhance the release of IL-1β and IL-6 by THP1 cell-derived macrophages in vitro, while PG meshes slightly reduce cytokine secretion. In this context, IL-1β and IL-6 are highly relevant as increased systemic levels of both cytokines are the main factors that stimulate the hepatic secretion of CRP [20, 21, 39]. In the same line, patients with UP meshes develop higher systemic CRP levels in vivo compared to patients with PG meshes. These data suggest that the implantation of UP meshes provokes a stronger inflammatory response compared to PG meshes.

In vitro, we investigated the effect of the hernia meshes on M0- and M1-like macrophages, because these types of macrophages are expected to be present early after surgery and are the main sources of IL-1β and IL-6.

Damage-associated molecular patterns originating from the surgically traumatized tissue and further stimuli originating from the implanted hernia meshes presumably activate monocytes/macrophages in vivo [7, 23, 40, 41]. This induces complex immunological processes, in which pro-inflammatory M1-like macrophages and the secretion of the pro-inflammatory IL-1β and the pro- and anti-inflammatory IL-6 are expected to play a major role [14, 22, 23]. To model this situation in vitro, monocytic THP-1 cells were differentiated towards M0-like macrophages or to pro-inflammatory M1-like macrophages in the presence or absence of UP or PG meshes. Cells were further stimulated with LPS to induce the expression and secretion of IL-6 and the expression of pro-IL-1β, an inactive cytoplasmic precursor of IL-1β [35, 42, 43]. In vivo diverse danger- or pathogen-associated molecular patterns originating from the damaged tissue or from invading pathogens can induce a primed state of macrophages similar to LPS [32]. Consecutive stimuli such as extracellular ATP or the pore-forming bacterial toxin nigericin induce the assembly of the NLRP3 inflammasome and the release of mature IL-1β [37]. Extracellular ATP released by damaged cells in vivo is a typical stimulus inducing sterile inflammation [36, 38]. UP meshes seemed to provide pro-inflammatory stimuli, since they enhanced the release of IL-1β and the release of IL-6 by M0-like macrophages. In contrast, PG meshes did not further stimulate cytokine release but slightly reduced the IL-6 secretion by M1-like macrophages.

These differences seen in vitro are in line with our finding that patients with UP meshes had significantly higher postoperative CRP levels compared to patients with PG meshes. Higher postoperative inflammation might also be associated with increased discomfort and pain [44, 45], which could not be investigated in this retrospective study. However, the long-term outcome of sublay herniotomy is of utmost importance and presumably depends on the degree of local perioperative inflammation. Although our findings from in vivo experiments as well as from our clinical cohort study does not prove definitive causality, ample evidence in the context of wound healing and foreign body reactions suggests that the macrophage reaction importantly influences the long-term outcome of sublay herniotomy [2, 3, 14, 19, 22, 23]. In addition to the immediate postoperative phase of acute inflammation dominated by M1-macrophages and the M2-dominated phase of scar formation, there is a third phase of wound healing characterized by resolution of the scar and reconstitution of a healthy steady state [46]. In the context of sublay herniotomy, however, this may cause hernia recurrence, because it might destabilize the integration of the hernia mesh into the surrounding tissue. We hypothesize that early – and possibly also chronic – postoperative inflammation plays a decisive role in determining the extent of tissue scaring and its resistance to resolution.

Hernia meshes continuously liberate pro-inflammatory factors including soluble degradation products and microparticles that might maintain the circle of infiltrating monocytes, their differentiation to macrophages, resulting in long-term hernia stabilization. In line with these hypotheses, the recurrence rate in the PG patient cohort tended to be higher compared to the UP cohort. However, this difference did not reach the level of statistical significance. A large-scale prospective clinical study is needed to clarify this important aspect.

We can only speculate, why UP hernia meshes provoke more inflammation compared to the PG meshes both in vitro and in vivo. As mentioned above, UP meshes are composites of non-absorbable polypropylene (Prolene^™^) and resorbable copolymers of epsilon-caprolactone and glycolide (Monocryl^™^). PG meshes are composed of a non-absorbable polyethylene terephthalate mesh with resorbable poly-lactic acid microgrips. While UP meshes are fixed on the surrounding tissue with a few stiches, PG meshes are self-fixating due to the presence of thousands of microgrips. These differences in the surgical techniques and the potential microlesions provoked by the surfaces of the meshes can account for the different inflammatory responses in vivo, but not for the differences in the experimental setting in vitro. The non-absorbable moieties of both meshes are polyesters with similar properties, which should not release relevant amounts of soluble compounds. The absorbable moieties, however, differ, releasing different compounds upon hydrolysis. Epsilon-caprolactone present in UP meshes degrades towards 6-hydroxycaproic acid, a fatty acid that is metabolized via the citric acid cycle [47]. The polyglycolic acid in UP meshes degrades to glycolic acid, which can be further metabolized to oxalic acid [47]. Depending on their local concentration, all three compounds can promote inflammation and oxalic acid can lead to formation of the insoluble calcium oxalate salt, which is a stimulus of inflammasome assembly and IL-1β release [48, 49]. By contrast, biodegradation of poly-lactic acid present in PG meshes releases L-lactic acid, a compound that can result in a metabolic reprogramming of macrophages [50]. In addition to the chemical differences among the meshes, microscopic and submicroscopic structural differences of the meshes may also play a role in different tissue reactions. UP has larger pores and a lighter weight than PG.

This study has several limitations. The in vitro cytokine release experiments reflect only one of many aspects of sterile inflammation and the potential involvement of adaptive immunity was not addressed. This simplified approach does not reflect the full complexity of the local and systemic immune response following mesh implantation. In addition to IL-1β and IL-6, other cytokines should be investigated in future more comprehensive studies. Furthermore, M2-like macrophages need to be investigated. M2-like macrophages, which predominantly secrete anti-inflammatory mediators and are expected to play a role during the phase of tissue remodeling and scar formation in the long-term, were not investigated in our study. We also do not provide mechanistic data to directly link the in vitro findings to the clinical observations. Although clear differences in cytokine release in vitro and systemic inflammation in vivo were observed, these did not translate into significant differences in postoperative clinical outcomes. This limits interpretability but highlights the multifactorial nature of the foreign body response in hernia surgery. The clinical part of this study has the typical limitations of a retrospective, single center analysis with limited sample sizes. Systemic IL-1β and IL-6 levels were not measured, and no patient-reported outcomes were available. Moreover, the two mesh types (UP and PG) were not used concurrently but in successive time periods. Meanwhile, changes occurred within the surgical team, and apart from the omission of mesh fixation in the PG group, subtle modifications in surgical technique may also have influenced outcomes.

Because of the numerous limitations, this study should be considered as a pilot study that does not allow for firm conclusions, but may serve as a basis for the generation of hypotheses and the design for future studies.

## Conclusions

Our in vitro and in vivo data suggest that implantation of UP meshes provoke a stronger perioperative inflammatory response compared to PG meshes. Reduced mesh-induced inflammation however, might lead to an increased recurrence rate, a highly relevant clinical concern. As a pilot study, these findings cannot be conclusively validated and warrant further investigation. Future in vitro studies should expand the range of inflammatory markers and include diverse immune cell types, particularly primary cells, to more accurately reflect physiological conditions. Larger rigorous, prospective randomized trials are essential to determine the extent to which mesh-induced perioperative inflammation influences clinical symptoms, patient-reported outcomes, systemic inflammation, tissue remodeling, scar formation, mesh integration, and ultimately hernia recurrence.

## Electronic supplementary material

Below is the link to the electronic supplementary material.

Supplementary Material 1: Patient allocation flow chart.

Supplementary Material 2: Supplement 2: perioperative white blood cell count in peripheral blood. Differences in white blood cell count in peripheral blood after hernia repair using the sublay technique between ULTRAPRO^®^ (UP) and ProGrip™ (PG) meshes. A: preoperative white blood cell count in peripheral blood. B: peak white blood cell count in peripheral blood on postoperative days (POD) 2 or 3. C: peak white blood cell count in peripheral blood until postoperative day 4. D: white blood cell count in peripheral blood at discharge. Data are presented as boxplots, where the boxes represent the interquartile range (25th to 75th percentiles), the horizontal line indicates the median, and whiskers show the minimum and maximum values. Comparisons between two groups were performed using the Mann–Whitney U test. Results are reported with the corresponding p-values, and *p* ≤ 0.05 was considered statistically significant.

Supplementary Material 3: Supplement 3: linear regression analysis of postoperative peak systemic C-reactive protein values or postoperative peak white blood cell counts and sizes of mesh implants. A-D: Peak C-reactive protein values on postoperative days (POD) 2 or 3 (A, B) or until postoperative day 4 (C, D) in patients who underwent hernia repair by sublay technique using either ULTRAPRO^®^ (UP) or ProGrip^™^ (PG) meshes. E-H: Peak white blood cell count on postoperative days (POD) 2 or 3 (E, F) or until postoperative day 4 (G, H) in patients who underwent hernia repair by sublay technique using either ULTRAPRO^®^ or ProGrip^™^ meshes.

## Data Availability

The datasets used and/or analyzed during the current study are available from the corresponding author on reasonable request.

## Abbreviations

ASA: Anesthesiologist´s classification of physical health score
CCI: Charlson Comorbidity Index
CRP: C-Reactive Protein
ICU: Intensive Care Unit
IL: Interleukin
POD: Postoperative Day
LPS: Lipopolysaccharide
ELISA: Enzyme-Linked Immuno Sorbent Assay
IFN-γ: Interferon-γ
PMA: Phorbol 12-Myristate 13-Acetate
ATP: Adenosine Triphosphate
