# Supplementary figures and images for: Acute inflammation triggered by two lightweight hernia meshes: a comparative in vitro and retrospective cohort study
Source: Hernia. 2025 Jun 17;29(1):205. doi: 10.1007/s10029-025-03391-y (PMC12174272; doi:10.1007/s10029-025-03391-y)

Supplement 1: Patient allocation flow chart.

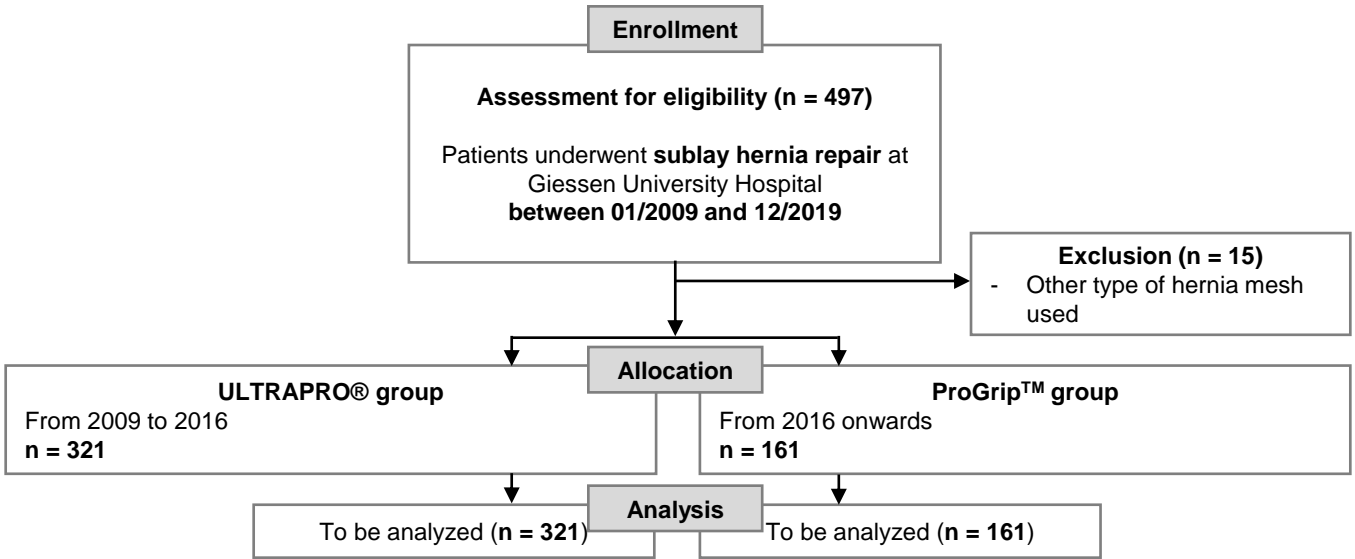

Supplement: Supplementary file 1 — Supplementary Material 1: Patient allocation flow chart. [file 10029_2025_3391_MOESM1_ESM.pdf]
