# Supplementary material for: Acute inflammation triggered by two lightweight hernia meshes: a comparative in vitro and retrospective cohort study
Source: Hernia. 2025 Jun 17;29(1):205. doi: 10.1007/s10029-025-03391-y (PMC12174272; doi:10.1007/s10029-025-03391-y)
Supplement: Supplementary file 2 — Supplementary Material 2: Supplement 2: perioperative white blood cell count in peripheral blood. Differences in white blood cell count in peripheral blood after hernia repair using the sublay technique between ULTRAPRO® (UP) and ProGrip™ (PG) meshes. A: preoperative white blood cell count in peripheral blood. B: peak white blood cell count in peripheral blood on postoperative days (POD) 2 or 3. C: peak white blood cell count in peripheral blood until postoperative day 4. D: white blood cell count in peripheral blood at discharge. Data are presented as boxplots, where the boxes represent the interquartile range (25th to 75th percentiles), the horizontal line indicates the median, and whiskers show the minimum and maximum values. Comparisons between two groups were performed using the Mann–Whitney U test. Results are reported with the corresponding p-values, and p ≤ 0.05 was considered statistically significant. [file 10029_2025_3391_MOESM2_ESM.pdf]

Acute inflammation triggered by two lightweight hernia meshes: a comparative *in vitro* and retrospective cohort study

Supplement 2: Perioperative white blood cell count in peripheral blood.

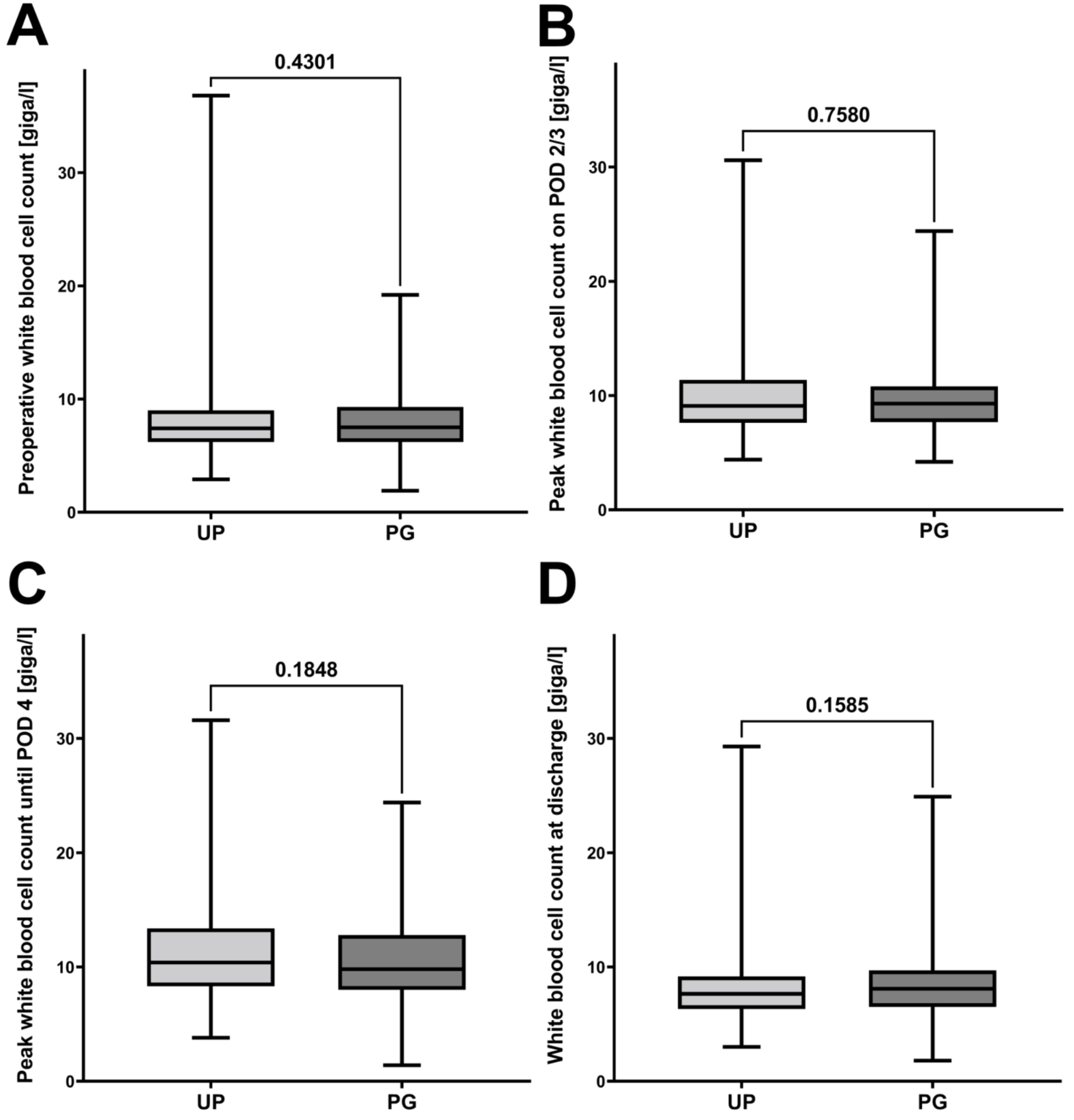

Differences in white blood cell count in peripheral blood after hernia repair using the sublay technique between ULTRAPRO® (UP) and ProGrip™ (PG) meshes. **A**: preoperative white blood cell count in peripheral blood. **B**: peak white blood cell count in peripheral blood on postoperative days (POD) 2 or 3. **C**: peak white blood cell count in peripheral blood until postoperative day 4. **D**: white blood cell count in peripheral blood at discharge. Data are presented as boxplots, where the boxes represent the interquartile range (25th to 75th percentiles), the horizontal line indicates the median, and whiskers show the minimum and maximum values. Comparisons between two groups were performed using the Mann–Whitney U test. Results are reported with the corresponding p-values, and $p \le 0.05$ was considered statistically significant.
