# Supplementary material for: Acute inflammation triggered by two lightweight hernia meshes: a comparative in vitro and retrospective cohort study
Source: Hernia. 2025 Jun 17;29(1):205. doi: 10.1007/s10029-025-03391-y (PMC12174272; doi:10.1007/s10029-025-03391-y)
Supplement: Supplementary file 3 — Supplementary Material 3: Supplement 3: linear regression analysis of postoperative peak systemic C-reactive protein values or postoperative peak white blood cell counts and sizes of mesh implants. A-D: Peak C-reactive protein values on postoperative days (POD) 2 or 3 (A, B) or until postoperative day 4 (C, D) in patients who underwent hernia repair by sublay technique using either ULTRAPRO® (UP) or ProGrip™ (PG) meshes. E-H: Peak white blood cell count on postoperative days (POD) 2 or 3 (E, F) or until postoperative day 4 (G, H) in patients who underwent hernia repair by sublay technique using either ULTRAPRO® or ProGrip™ meshes. [file 10029_2025_3391_MOESM3_ESM.pdf]

Supplement 3: Linear regression analysis of postoperative peak systemic C-reactive protein values or postoperative peak white blood cell counts and sizes of mesh implants.

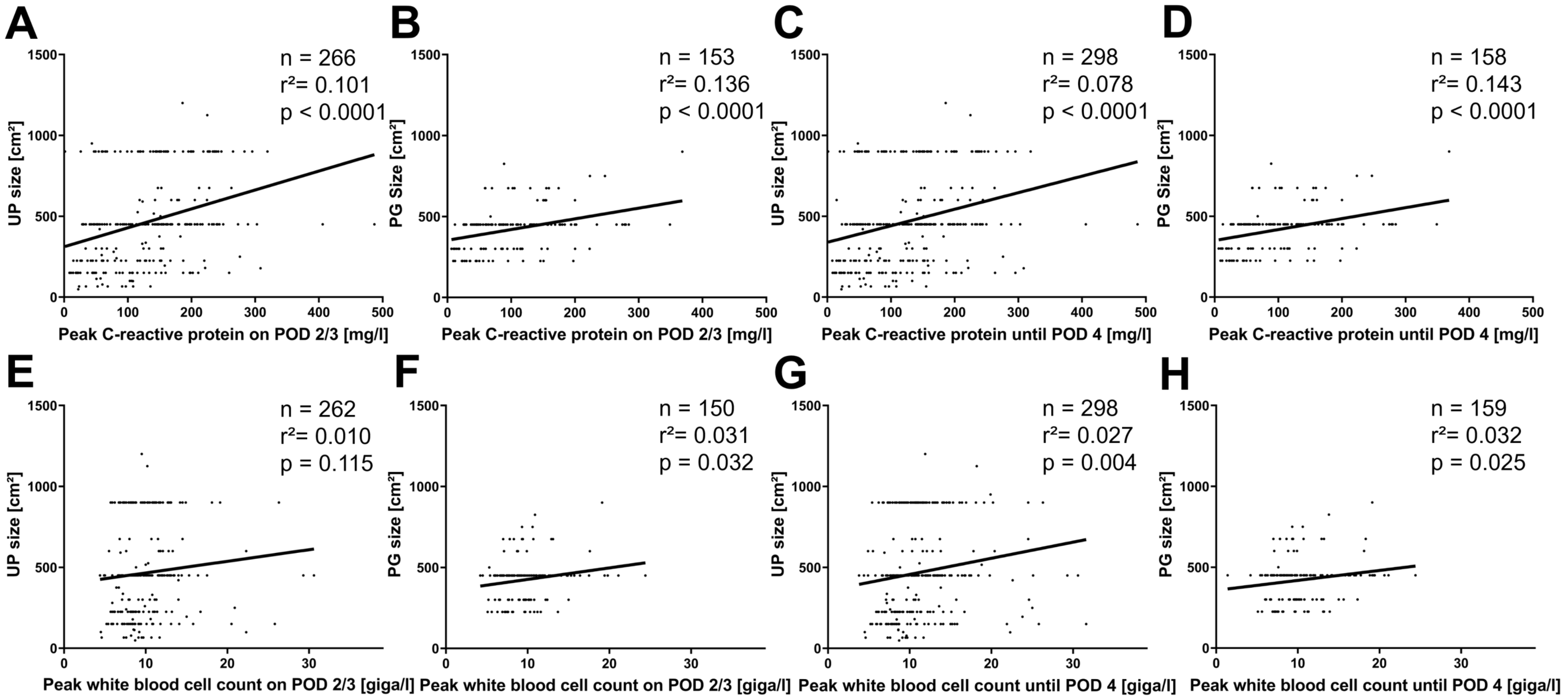

A-D: Peak C-reactive protein values on postoperative days (POD) 2 or 3 (**A, B**) or until postoperative day 4 (**C, D**) in patients who underwent hernia repair by sublay technique using either ULTRAPRO® (UP) or ProGrip™ (PG) meshes. **E-H**: Peak white blood cell count on postoperative days (POD) 2 or 3 (**E, F**) or until postoperative day 4 (**G, H**) in patients who underwent hernia repair by sublay technique using either ULTRAPRO® or ProGrip™ meshes.
